# Factors Associated With the Health and Economic Effects of the COVID-19 Pandemic in the Peruvian Textile Sector, 2020–2021

**DOI:** 10.3389/fsoc.2022.875998

**Published:** 2022-04-29

**Authors:** Juan Arroyo-Laguna, Raúl Timaná-Ruíz

**Affiliations:** ^1^Postgraduate School, Saint Ignatius of Loyola University, Lima, Peru; ^2^Integral Health Insurance, Ministry of Health, Lima, Peru

**Keywords:** COVID-19 pandemic, health impact, economic impact, quarantine, textile industry

## Abstract

The article identifies the factors associated with the health and economic effects of the COVID-19 pandemic on people working in the textile industry of Lima, Peru, during 2021. The study was conducted in Peru's largest textile emporium, so-called Gamarra. The study design is observational and cross-sectional, with two models with two temporal samples for the first and second waves of the COVID-19 pandemic. The first model measures the chance of getting sick from COVID-19. The second model measures the economic impact by the variations in incomes. Inferential statistics are employed, using the chi-square test. The *p*-value (*p* < 0.05) is evaluated to decide the statistical significance of the variables. Of 820 workers included, 48% work in street trading, 45% are ≤ 35 years of age and 15% are foreign migrants. Logistic regression analysis for the first model reveals an association between infection by a family member, people breaking quarantine, foreign nationality, not having hygienic services and having a chronic disease, with the highest probability of COVID-19 infection. Regarding economic impact, an association is found between educational level, being ≥45 years of age and infection of a family member, with a greater probability of variation in income.

## Introduction

Humankind has seen irreparable consequences of the COVID-19 pandemic; up until this moment in time, more than 5 million people have died and more than 250 million cases have been confirmed around the world (World Health Organization, 2021). The pandemic has led countries to adopt different health measures to contain the virus, considering its level of contagiousness and lethality (Zheng et al., 2020). These measures have led to important health, economic, and social repercussions, and this period has been referred to as the “new normal” (Mitchell and Nou, 2020).

In Peru, more than 200 thousand deaths and more than 2 million cases of SARS-COV-2 have been reported (Sala Situacional COVID-19 Perú, 2021). This occurred in two waves, which were fully identified by the Peruvian health authorities: the first wave occurred between March and November 2020 and the second wave occurred between December 2020 and May 2021. Within this context, the Peruvian health system, immersed in a long-standing precarious situation, collapsed due to the patients' high health demand that required critical health units and oxygen (Ponce de León, 2021).

The economic consequences were also devastating. Peru is a country known for its great informality; it is estimated that 35%−45% of GDP and 60% of the hours worked come from the informal economy (Machado, 2014; Arroyo, 2020). In this regard, in Peru, a mandatory quarantine was decreed due to the COVID-19 pandemic to contain contagion and mortality (Huamaní et al., 2020). However, this had calamitous economic effects on the informal sector, which depends on daily and face-to-face sales of its products and services (Jaramillo and Ñopo, 2020a; Barrutia Barreto et al., 2021).

The COVID-19 pandemic has impacted individuals, societies, and countries in a variety of ways; however, there are certain factors that may determine the increased likelihood of greater health or economic impact (Abrams and Szefler, 2020). Several associated factors have been described such as, the presence of chronic diseases, lack of basic services, ethnicity, immigration status, socioeconomic level, and overcrowding, that increase the probability of causing a greater impact of COVID-19 on people (Burström and Tao, 2020; Rollston and Galea, 2020).

Countries such as India and others in South Asia have reported that one of the economic sectors most affected by the COVID-19 pandemic has been the textile sector or industry (Majumdar et al., 2020; Kaur, 2021). Quarantines have affected production, the supply chain, and the price of raw materials, and have led to the loss of skilled human resources through contagion or death (Chakraborty and Chandra Biswas, 2020). In Peru, the most important textile commercial sector is the so-called “Gamarra” located in the capital Lima in the district of La Victoria, which comprises more than 39 thousand companies with nearly 80 thousand workers distributed in small overcrowded and slum spaces that congregate migrants from many provinces looking for a job opportunity in the capital (Sihuacuyo Rodríguez and Arisaca Llanque, 2017). In this sense, this study identifies the determinants of the health and economic impact of the COVID-19 pandemic on people working in the commercial textile sector of Gamarra.

## Materials and Methods

The study design was observational and cross-sectional. The population comprised people working in the commercial center of Gamarra in the District of La Victoria in Metropolitan Lima. The people included in this study were over the age of 18, working somewhere in the Gamarra commercial center, who agreed to participate in the study by signing the informed consent form. People under 18 years of age, those who did not complete the entire survey, and those who were not working at the time of the study were excluded.

Two models were designed for this study with two time samples each for the first wave (March–November 2020) and the second wave (December 2020–May 2021). The dependent variable for the first model was if the person got sick with COVID-19, and for the second model was the change in income. The change in income was reported in five categories from “decreased a lot” to “increased a lot” on a Likert scale. The COVID-19 contagion variable was reported as a dichotomous categorical variable.

The model was built based on a review of the literature on factors associated with the health and economic effects of the COVID-19 pandemic. The available evidence showed that, since it is an ongoing pandemic, the conceptual models are still under construction. In this sense, a *de novo* construction of a conceptual framework was carried out based on the available evidence and the judgment of experts. The review of the literature also showed that there is a greater number of documents related to the factors associated with the risk of getting sick from COVID-19, in contrast to the scarce evidence on the factors associated with the probability of variation in people's economic income.

Based on the conceptual model resulting from the review of the literature for the first model, the independent variables that were considered were informality (dependent, independent, owners), nationality, type of work (ambulant, trader, producer or garment maker), access to basic services, presence of chronic disease, contagion from a family member and overcrowding (Khalatbari-Soltani et al., 2020; Sá, 2020; Choi et al., 2021). The researchers considered, based on the Peruvian context, adding the variables compliance with quarantine and the number of relatives who carry out face-to-face work as explanatory variables of the health impact. For the second model, the independent variables were educational level, sex, informality, nationality, having contracted COVID, age (categorized according to age ranges) and the presence of severe COVID-19 cases in the close family of the respondent (understood as those who needed medical care financed by the respondents), according to the review of the literature (Guerrero Ble and Graham, 2020; Jaramillo and Ñopo, 2020a,b,c). In addition, the researchers, according to the Peruvian context, added the variable access to economic aid from the central government.

The sample was calculated using the formula for known population, identifying the reference size of 784 workers in Gamarra as the proportion of the population that has the characteristic of interest (50%), confidence level (95%), and estimation error (5%). In addition, an expected proportion of losses or non-response of 10% was considered, hence, the final sample was 852 people working in Gamarra. The sampling technique was random sampling by quota sampling.

A 26-item, multiple-choice, survey-type questionnaire was developed as an instrument divided into seven sections, including questions from both waves on sociodemographic aspects, information on economic activity, income variation, coronavirus contagion, and measures to deal with COVID-19. The survey was developed *ad hoc* for this study considering the limited information available in the world to answer our research question. The instrument was validated by a public health expert and an epidemiology expert.

Between June and July 2021, the entire instrument was applied to each participant, running between two and five days per month, between 09:00 and 18:00 h. The survey locations were places characterized by high business concentration. During the fieldwork, the safety of both the surveyors and the workers was guaranteed, in compliance with the biosecurity measures dictated by the Peruvian government.

The surveyors were two management and senior management students, who were familiar with the instrument. The surveyors received training on the objectives and variables of the study, as well as on the correct filling out of the data collection forms. In addition, a pilot test was conducted with 10 workers prior to the start of the fieldwork.

The data obtained from the surveys were coded and recorded in a database in Microsoft Excel 2013 program and underwent a data quality review; for this purpose, two data entry clerks were hired who separately entered the information into the program. Subsequently, two of the authors checked both files to verify the correspondence of the data entered.

The statistical analysis was performed using the STATA statistical software version 14. Descriptive statistics were used for categorical variables, such as the variation in income and getting sick from COVID-19, which were reported as frequencies and percentages. To evaluate the association between the categorical variables of the study, inferential statistics were employed, using the chi-square test. The *p*-value (*p* < 0.05) was evaluated to decide the statistical significance of the variables.

Logistic regression analysis (logit and ordered logit) was also performed for each of the models, where the outcome variable was health impact for the first model and economic impact for the second. The predictor variables for the first model were informality, nationality, type of work, chronic disease, access to basic services, overcrowding, compliance with quarantine, infection by a family member, and the number of family members doing on-site work. For the second model, the predictor variables were educational level, gender, informality, nationality, having contracted COVID, access to economic support from the central government, cases of severe COVID-19 in the respondent's close family, and age. An analysis of correlations between the variables was performed to determine whether there was autocorrelation of the variables in each of the models. Odds ratio (OR) with its 95% confidence interval (95%CI) was used as a measure of association and the *p*-value (*p* < 0.05) was evaluated to decide the statistical significance of the variables in each of the models. The crude models were tested for fitness according to the available evidence. The study was approved by the Institutional Ethics Committee of the University. All participants signed the written informed consent in accordance with the Declaration of Helsinki.

## Results

The study included 820 workers in Gamarra, ranging in age from 18 to 70 years old. A total of 15% were between 18 and 24 years old, 30% were between 25 and 34 years old, 23% were between 35 and 44 years old, and 32% were over 45 years old. Moreover, 52% were women and 15% were Venezuelan. In addition, 48% were classified as traders, 25% as producers or garment manufacturers, and 27% as store merchants. Of those surveyed, 61% said that they had not received a government bonus, 53% said that they did not have health insurance, and 89% said they did not have a chronic disease. Likewise, 89% reported that they had undergone quarantine (Table 1).

**Table 1:** Characteristics of the Gamarra workers included in the study (*n* = 820).

**Characteristics**	**No**.	**%**
Gender		
Women	422	51%
Men	398	49%
Age		
18–24	122	15%
25–34	243	30%
35–44	192	23%
45–54	142	17%
55–70	113	14%
More than 70 years	8	1%
Nationality		
Bolivian	1	0%
Peruvian	696	85%
Venezuelan	123	15%
Educational level		
University Post-Graduate	6	1%
Completed Elementary school/High school incomplete	127	15%
Completed high school/Technical college incomplete	381	46%
Not educated/Initial education/Primary school incomplete	121	15%
Completed technical college	111	14%
Tertiary/university education incomplete/complete	74	9%
Type of work		
Trader	392	48%
Store merchant (non-employee)	16	2%
Store merchant (owner)	49	6%
Store merchant (employee)	154	19%
Producer or manufacturer	209	25%
Grant beneficiary		
No	503	61%
Yes	317	39%
Has chronic disease		
No	728	89%
Yes	92	11%
Has health insurance		
No	438	53%
Yes	382	47%
Carried out quarantine		
No	89	11%
Yes	731	89%
Infected in first wave		
No	600	73%
Yes	220	27%
Infected in second wave		
No	719	88%
Yes	101	12%
Income variation during first wave		
Increased	4	0%
Increased a lot	1	0%
Decreased	120	15%
Decreased a lot	672	82%
No variation	23	3%
Income variation during second wave		
Increased	227	28%
Increased a lot	1	0%
Decreased	217	26%
Decreased a lot	181	22%
No variation	194	24%

A total of 27% and 12% of the respondents reported that they were infected in the first and second wave, respectively. Additionally, 15% and 82% reported that their income decreased or decreased a lot in the first wave, respectively. Only 3% said that their income did not vary during the first wave. For the second wave, only 26% and 22% reported that their income decreased or decreased a lot, respectively, whereas 28% said that their income increased and 24% said that it remained unchanged (Table 1).

The bivariate analysis shows a statistically significant association between getting sick from COVID-19 in the first wave and informality, having hygienic services and contagion from a family member. In the second wave, the variables quarantine and infection of a family member were statistically significant (Table 2).

**Table 2:** Bivariate analysis between associated factors and get sick from COVID-19 in the first and second waves (*n* = 820).

**Determinants**	**First wave (*****n*** **=** **820)**			**Second wave (*****n*** **=** **820)**		
	**Infected**	**Not infected**	***p* value**	**Infected**	**Not infected**	***p* value**
	***n*** **(%)**	***n*** **(%)**		***n*** **(%)**	***n*** **(%)**	
Nationality			0.419			0.348
Bolivian	1	0		1	0	
Peruvian	191	505		83	613	
Venezuelan	28	95		17	106	
Type of activity			0.724			0.692
Trader	109	283		45	347	
Store merchant (non-employee)	5	11		2	14	
Store merchant (owner)	11	38		13	36	
Store merchant (employee)	38	116		15	139	
Producer or manufacturer	57	152		26	183	
Quarantine			0.723			0.04
Yes	188	543		89	642	
No	32	57		12	77	
Sanitary services			0.006			0.555
Shared bathroom outside of living space	46	137		15	168	
Bathroom within living space	170	457		82	545	
No bathroom/Not connected to public grid	4	6		4	6	
Water			0.181			0.681
Has shared water service	42	129		14	157	
Has water service within living space	169	451		83	537	
No water service	9	20		4	25	
Chronic disease			0.01			0.115
Yes	31	61		19	73	
No	189	539		82	646	
Family member infected			0.905			0.02
Yes	33	23		25	18	
No	187	577		76	701	
Informality			0.035			0.666
Yes	176	488		74	590	
No	44	112		27	129	

The variation in income in the first wave was significantly associated with educational level, nationality, and having received a bonus from the central government, while that in the second wave had statistically significant differences with the variable educational level and contagion from a family member.

The logistic regression analysis for the variation in income and the probability of becoming ill did not present autocorrelation of variables. The model that evaluated whether the person became ill with COVID-19 in the two waves was adjusted for the variables infection by a family member and the number of family members doing face-to-face work (Otero, 2020), showing a better fit than the first model. The model that evaluated the income variation was adjusted by the variable age (CELADE, 2020), showing a better fit for the first wave, but for the second wave, the fit was lower in the first model.

The logistic regression analysis showed that in the first wave, infection by a family member (OR: 4.44; 95%CI: 2.51–7.84) and failure to observe quarantine (OR: 1.72; 95%CI: 1.06–2.79) had a higher probability of becoming ill from COVID-19. The other variables were not statistically significant. In the second wave, foreign nationality (OR: 2.16; 95%CI: 1.13–4.12), not having sanitary facilities (OR: 17.79; 95%CI: 1.61–196.98), having a chronic disease (OR: 2.17; 95%CI: 1.16–4.04) and infection by a family member were more likely to be infected by COVID-19 (OR: 14.21; 95%CI: 7.13–28.31); the other variables were not statistically significant (Table 3).

**Table 3:** Factors associated with becoming ill with COVID-19 in the first and second waves (*n* = 820).

**Variables**	**First wave**				**Second wave**			
	**Adjusted OR**	***p* value**	**IC95%**		**OR Adjusted**	***p* value**	**CI95%**	
			**Lower**	**Higher**			**Lower**	**Higher**
Informality	0.88	0.571	0.56	1.37	0.63	0.134	0.34	1.15
Foreign	0.82	0.426	0.50	1.34	2.16	0.019	1.13	4.12
Store merchant	0.80	0.324	0.51	1.25	1.10	0.76	0.59	2.04
Street merchant	0.97	0.884	0.63	1.49	1.02	0.954	0.54	1.93
Quarantine non-compliance	1.72	0.028	1.06	2.79	1.14	0.723	0.56	2.32
Overcrowding	0.84	0.546	0.48475	1.47	1.69	0.142	0.84	3.39
Bathroom within living space	0.91	0.851	0.36	2.33	1.83	0.411	0.43	7.76
No bathroom/Not connected to public grid	0.87	0.877	0.15	5.11	17.79	0.019	1.61	196.98
Water service within living space	1.13	0.805	0.43	2.98	1.24	0.777	0.28	5.55
No water service	1.40	0.605	0.39	5.05	0.56	0.593	0.07	4.72
Chronic disease	1.33	0.248	0.82	2.16	2.17	0.015	1.16	4.04
Family members who work in person	1.01	0.862	0.89	1.15	0.89	0.258	0.73	1.09
Family member infected in the PO	4.44	0	2.51	7.84	14.21	0	7.13	28.31

In the first wave, having incomplete or complete university education (OR: 2.41; 95%CI: 1.08–5.34), being a foreigner (OR: 1.76; 95%CI: 1.05–2.97), and people with age ranging 45–54 years (OR: 0.41; 95%CI: 0.21–0.79) and 55–70 years (OR: 0.41; 95%CI: 0.19–0.86) had more probability of variation in income according to statistical regression analysis; the other variables were not statistically significant. In the second wave, the level of education and infection by a family member showed a greater probability for the variation in income (OR: 0.46; 95%CI: 0.25–0.85); the other variables were not statistically significant (Table 4).

**Table 4:** Factors associated with income variation in the first and second waves (*n* = 820).

**Variables**	**First wave**				**Second wave**			
	**Adjusted OR**	***p* value**	**IC95%**		**OR Adjusted**	***p* value**	**CI95%**	
			**Lower**	**Higher**			**Lower**	**Higher**
Completed Elementary school/High school incomplete	1.46	0.34	0.67	3.20	1.70	0.023	1.07	2.70
Completed high school/Technical College incomplete	1.72	0.085	0.93	3.21	3.33	0	2.27	4.88
Technical college complete	1.54	0.264	0.72	3.29	3.21	0	1.99	5.19
Tertiary/university education complete or incomplete	2.41	0.031	1.08	5.34	5.62	0	3.24	9.76
University Post-graduate	-	0.99	-	-	5.16	0.037	1.10	24.25
Gender	1.34	0.124	0.92	1.94	1.03	0.821	0.80	1.32
Informality	0.84	0.478	0.52	1.36	0.80	0.192	0.57	1.12
Foreign	1.76	0.033	1.05	2.97	0.80	0.264	0.55	1.18
Infected PO	0.93	0.731	0.60	1.43	1.11	0.619	0.73	1.68
Grant	0.85	0.461	0.56	1.30	0.85	0.254	0.64	1.12
Family member infected PO	1.16	0.681	0.57	2.39	0.46	0.012	0.25	0.85
25–34	0.63	0.08	0.37	1.06				
35–44	0.58	0.054	0.33	1.01				
45–54	0.41	0.008	0.21	0.79				
55–70	0.41	0.019	0.19	0.86				
More than 70 years	-	0.988	-	-				

## Discussion

The factors associated with a higher probability of getting sick from COVID-19 among Gamarra workers were the infection of a family member, failure to comply with quarantine, foreign nationality, lack of sanitary services, and having a chronic disease in both the first and second waves. These findings are compatible with other studies conducted internationally (Oronce et al., 2020; Qiu et al., 2020; Amato et al., 2021; Doblhammer et al., 2021); however, these studies were conducted in the general population as the unit of analysis or with specific regions or areas of some countries. Some local studies have identified several factors related to increased mortality from COVID-19 (Hueda-Zavaleta et al., 2021; Yupari-Azabache et al., 2021). In this sense, we did not find local antecedents that aim to evaluate the determinants or factors associated with the increase in COVID-19 infection in Peru, especially in the textile trade sector, with high levels of informality, which is why we consider the findings of this study to be relevant. However, two bibliographic references were found at the local level that used methods similar to our study; however, the explanatory variable referred to the area of mental health and the population corresponded to health workers (Yáñez et al., 2020; Zhang et al., 2020). The possible explanations for the differences found with international research could be due to the fact that the population studied could have distinctive characteristics from the general population, as it belongs to a working textile trade sector with social and occupational dynamics related to informality that could be more relevant to increasing the probability of infection by COVID-19.

International studies have identified several factors related to COVID- infection. Varkey et al. (2020) reported in an analysis of the socioeconomic determinants of COVID-19 in Asian countries that the most important factors for the increase in COVID-19 cases were gross income per capita, migration, and social capital in each country. Al Kindi et al. (2021) found that the most important factors for COVID-19 infection rates were migrant status, population density, the number of hospital beds, the number of households, and purchasing power. A study in Canada reported that COVID-19 infections are higher in communities with a greater proportion of black and low-income residents (Choi et al., 2021). These results show that there are other variables that could explain the increase in COVID-19 infection rates, such as race or income level; however, these dimensions were not addressed by this study.

Regarding the data on economic income variation, this study reports that in the first wave, 97% respondents mentioned that their income decreased or decreased a lot, compared to the second wave where only 48% reported that their income decreased or decreased a lot and 28% mentioned that their income increased. These results are consistent with a local study that concluded the presence of some economic problems in several aspects of the family economy (Barrutia Barreto et al., 2021); however, it does not report absolute or relative frequency of income variation. Additionally, most studies that aim to quantify or explain the economic consequences address the macroeconomic perspective of the country, rather than reporting data that help us to compare our results with local research (Cuenca Jaque et al., 2020; Olivera Cáceres and Loza Ticona, 2021; Trujillo Figueroa and Mendoza Briceño, 2021). However, an unpublished local survey mentions that quarantine affected 77% of Peruvian households, reporting that their income decreased by 68%, and 70% accumulated debts in the COVID-19 pandemic (Fuentes, 2020).

The factors associated with the variation in income during the COVID-19 pandemic in this study were being a foreigner, having any level of education, being 45–70 years of age, and being infected by a family member. We did not find similar studies at the local level that could contrast these data. However, Jaramillo and Ñopo (2020a) analyze the economic impact of the COVID-19 pandemic on families from the perspective of labor income, reporting the vulnerability of households with informal jobs. However, the study does not quantify incomes, due to the difficulty of the respondents to make their income explicit in the midst of the health crisis, although they did allow us to know the percentage variation in income with respect to the previous situation.

International studies show the economic effects of the COVID-19 pandemic from a macroeconomic perspective. They report the multiple economic consequences of the COVID-19 pandemic on countries, highlighting the drastic decline in GDP, disruption of payment chains and exports, decline in employment, and deepening poverty (Ahmad et al., 2020; Ashraf, 2020; Sarkodie and Owusu, 2020). Additionally, other studies have investigated income distribution and its variability in times of COVID-19 pandemic. These studies indicate that the poorest, most vulnerable, and lower income sectors are the most affected in this pandemic, deepening inequality, poverty, and lack of opportunities (Albert et al., 2020; Baena-Díez et al., 2020; Li et al., 2020; Lustig et al., 2020; Gómez Bengoechea, 2021). A study in Argentina assessed the consequences of the COVID-19 pandemic on income, reporting that 96.1% believe that quarantine will affect their family economy, specifying that 41.1% mention that it will affect it a lot and 36.1%, quite a lot (Actis Di Pasquale et al., 2020).

In this sense, we consider that the perspective of this study and its findings contribute to understanding the variation in economic income of families in the textile trade sector in a country considered one of the most affected country by COVID-19 (Médicos Sin Fronteras, 2021). These findings could be due to the growing informality in this sector, which is why, after the decision to withdraw the quarantine measure, the perception of income variation changed ostensibly. This is in line with the perception that migrants are considered vulnerable and that most of them belong to the informal sector. In addition, the infection of a family member with COVID-19 can lead to an increase in out-of-pocket expenses and catastrophic health expenses and could additionally condition the care of this family member with the consequent loss of productivity and, therefore, decrease in income.

As limitations of the study, it should be mentioned that there were important variables that were not addressed considering that the evidence supporting them was published later, for instance, mortality and socioeconomic level. The cross-sectional design of the study at the end of the second wave might not capture significant differences in each wave of infection separately. In addition, the lack of economic evidence on the determinants of variation in income could influence the lack of other variables of interest in the regression model, likewise, the study takes as a sample Gamarra workers who had or did not have COVID-19 and did not capture those who at the time of the interview could be with COVID-19, this could be a study bias.

In conclusion, it was possible to demonstrate that there are variables such as infection by a family member, failure to comply with quarantine, foreign nationality, lack of sanitary services, chronic illness, education, and age that can explain infection and the variation in income during the COVID-19 pandemic in people working in the textile trade sector of Gamarra in Lima, Peru. Studies should be conducted to corroborate these findings, increase the number of variables in the respective models, and complement these data with qualitative information.

In summary, there are no similar studies in Peru. Internationally, studies on the effects of COVID-19 reveal a series of associated and explanatory factors of its impact, which are not comparable with ours because we focus on an informal target population, representative of 85% of the Peruvian economy and working population. Herein resides its relevance.

## Data Availability Statement

The raw data supporting the conclusions of this article will be made available by the authors, without undue reservation.

## Author Contributions

JA-L designed the research, methodology, conducted the research, analyzed the database, and wrote the article. RT-R was in charge of the analysis plan and first draft of results. Both authors approved the final version of the article.

## Funding

Saint Ignatius of Loyola University gave institutional backing to the team and pays for its publication. Fondo Nacional de Ciencia y Tecnología (FONDECYT - National Science and Technology Fund) paid for the study.

## Conflict of Interest

The authors declare that the research was conducted in the absence of any commercial or financial relationships that could be construed as a potential conflict of interest.

## Publisher's Note

All claims expressed in this article are solely those of the authors and do not necessarily represent those of their affiliated organizations, or those of the publisher, the editors and the reviewers. Any product that may be evaluated in this article, or claim that may be made by its manufacturer, is not guaranteed or endorsed by the publisher.
